# Increased adhesion of *Plasmodium falciparum* infected erythrocytes to ICAM-1 in children with acute intestinal injury

**DOI:** 10.1186/s12936-016-1110-3

**Published:** 2016-02-01

**Authors:** James A. Church, Lydia Nyamako, Peter Olupot-Olupot, Kathryn Maitland, Britta C. Urban

**Affiliations:** Centre for Paediatrics, Blizard Institute, Queen Mary University of London, London, UK; KEMRI-Wellcome Trust Research Programme, Kilifi, Kenya; Mbale Regional Referral Hospital Clinical Research Unit (MCRU), Mbale, Uganda; Wellcome Centre for Clinical Tropical Medicine, Imperial College, London, UK; Parasitology Department, Liverpool School of Tropical Medicine, Liverpool, UK; Busitema University Faculty of Health Sciences, Mbale Campus, Mbale, Uganda

**Keywords:** Malaria, Cytoadhesion, Bacteraemia, Intestinal injury, Endotoxin, Biomarkers

## Abstract

**Background:**

Children with severe malaria are at increased risk of invasive bacterial disease particularly infection with enteric gram-negative organisms. These organisms are likely to originate from the gut, however, how and why they breach the intestinal interface in the context of malaria infection remains unclear. One explanation is that accumulation of infected red blood cells (iRBCs) in the intestinal microvasculature contributes to tissue damage and subsequent microbial translocation which can be addressed through investigation of the impact of cytoadhesion in patients with malaria and intestinal damage.

**Methods:**

Using a static adhesion assay, cytoadhesion of iRBCs was quantified in 48 children with malaria to recombinant proteins constitutively expressed on endothelial cell surfaces. Cytoadhesive phenotypes between children with and without biochemical evidence of intestinal damage [defined as endotoxemia or elevated plasma intestinal fatty acid binding protein (I-FABP)] was compared.

**Results:**

The majority of parasites demonstrated binding to the endothelial receptors CD36 and to a lesser extent to ICAM-1. Reduced adhesion to CD36 but not adhesion to ICAM-1 or rosetting was associated with malarial anaemia (p = 0.004). Increased adhesion of iRBCs to ICAM-1 in children who had evidence of elevated I-FABP (p = 0.022), a marker of intestinal ischaemia was observed. There was no correlation between the presence of endotoxemia and increased adhesion to any of the recombinant proteins.

**Conclusion:**

Increased parasite adhesion to ICAM-1 in children with evidence of intestinal ischaemia lends further evidence to a link between the cytoadherence of iRBCs in gut microvasculature and intestinal damage.

## Background

Children with severe *Plasmodium falciparum* malaria are at increased risk of concomitant invasive bacterial infection (IBI) particularly with enteric gram-negative organisms (EGNOs) [1]. In a systematic review compiling data from epidemiological studies and clinical studies of paediatric hospital admissions of *P. falciparum* malaria (describing IBI) across sub-Saharan Africa, the mean prevalence of IBI co-infection was 6.4 % (95 % CI 5.81 −6.98 %). Bacterial co-infection results in higher case fatalities compared to children with severe malaria alone (24.1 versus 10.2 %). An estimated one-third of all severe malaria deaths in African children are attributable to bacterial co-infection [2]. The precise mechanism by which children with malaria are predisposed to bacteraemia are uncertain and is a major limitation in the development of new strategies to reduce morbidity and mortality in this disease.

The preponderance of gram-negative bacteraemias, including non-typhoidal salmonellae and *Escherichia coli*, in children with malaria is highly suggestive of an intestinal process involving translocation of intraluminal microbes across a compromised gut barrier. There is patchy evidence, which attests to gut barrier damage in malaria disease. Adults with acute malaria have reduced absorptive capacity as well as increased intestinal permeability measured using a differential sugar absorption test, the highest ratios being noted in severe disease [3]. Up to 50 % of Nigerian infants with uncomplicated malaria have gastrointestinal disturbances [4]. In addition, we previously showed that approximately 30 % of children admitted to hospital with malaria suffered from endotoxemia [5]. Endotoxin, a complex lipopolysaccharide (LPS), which is constituent in the cell walls of gram-negative bacteria, has been used in a variety of cohorts as a measure of microbial translocation [6, 7]. In 59 (29 %) children, raised plasma concentration of intestinal fatty acid binding protein (I-FABP), a marker of acute gut injury, were detected although elevated I-FABP and endotoxemia were not associated.

There are several mechanisms, not necessarily mutually exclusive, that might impair gut barrier integrity and function in children with malaria. Firstly, hypoperfusion may occur in the context of severe malarial anaemia and shock; the resulting hypoxic damage to the intestinal epithelium can lead to increased permeability with subsequent translocation of intestinal contents [8]. Secondly systemic inflammation, which accompanies sepsis as well as severe forms of *P. falciparum* malaria, can result in increased intestinal permeability even in the absence of enterocyte damage [9]. This may in part be mediated by l-arginine deficiency, a hallmark of malaria parasite infection, which has been shown to potentiate intestinal inflammation and permeability in mice [10]. Thirdly, an important factor in the pathogenesis of *P. falciparum* malaria is thought to be the adherence of infected red blood cells (iRBCs) to receptors on small vessel endothelial cells. The resultant sequestration of iRBCs in specific organs is most notable in the case of cerebral malaria where iRBCs accumulate in brain microvasculature. Moreover, findings from histopathological sections taken at autopsy from children who died of severe malaria suggest that intense sequestration is also manifest in the gut [11, 12]. The accumulation of iRBCs in the intestinal microvasculature is, therefore, another possible cause of tissue damage leading to microbial translocation.

To further explore the contribution of parasite sequestration to microbial translocation in the gut, parasite adhesion in children with both malaria and endotoxemia or acute gut injury was assessed. Adhesion was quantified in vitro by measuring rosetting of iRBCs with uninfected erythrocytes as well as their binding to receptors constitutively expressed on endothelial cell surfaces.

## Methods

### Study population

The study population has been described in detail elsewhere [5]. In brief, 257 children admitted to Mbale Regional Referral Hospital in Uganda with a diagnosis of malaria were recruited into the study. Based on severity of clinical symptoms, children were classified into those hospitalized with malaria (positive blood film and rapid diagnostic test) but without life-threatening clinical syndromes and those with severe malaria and at least one life-threatening clinical syndrome (Hb ≤5 dg/ml; impaired consciousness defined as prostration on clinical examination or coma; deep breathing) according to recruitment criteria for the FEAST trial (ISRCTN69856593) ongoing at that time [13]. A proportion of these children with severe malaria and signs of a life-threatening syndrome also had signs of impaired perfusion (defined as a capillary refill time of 3 or more seconds, lower-limb temperature gradient, weak radial-pulse volume, or severe tachycardia), and were recruited into the FEAST trial [13]. On admission, a 5 ml venous blood sample was taken and PBMCs, RBCs and plasma separated and stored at minus 80 °C.

The study was approved by the Ugandan Ethical Review Committee (REIRC 002/2009). Written informed consent was provided by all parents or guardians of children recruited into the study.

### Elisa

The plasma concentrations of TNF (R&D Systems, Inc.) and I-FABP (Hyglos GmbH) were determined using ELISA according to the manufacturer’s recommendations.

### EAA assay

An endotoxin activity assay, EAA™ (Spectral Diagnostics Inc, Toronto, Canada) was used to quantify endotoxin levels in EDTA blood, carried out within 3 h of venepuncture according to the manufacturer’s recommendation. A cut off value of 0.4 EAA units for clinically relevant endotoxemia was used, as evaluated in the MEDIC trial [6].

### Static adhesion assay and rosetting

Samples were blinded and remained so until all the data had been collected and analysed. RBCs were thawed and *P. falciparum* iRBCs cultured to maturity according to standard procedures [14]. Mature trophozoite stages were observed after a median time of 30 h (range 26–50 h). Cultures with a parasitaemia above 0.5 % were included and those with greater than 3 % parasitaemia were diluted down to 3 % (median parasitaemia 1 %, range 0.5–4.7 %). The laboratory line ITG selected for adhesion to ICAM-1, was used as a positive control for each experiment. When most of the parasites had reached the pigmented trophozoite stage, the culture was harvested and washed twice in binding medium (RPMI 1640 with 20 mM Hepes).

Recombinant ICAM-1-Fc (donated by Alister Craig’s laboratory) and CD36-Fc (R&D Systems Inc.) have been used before in static adhesion assays. Two less recognized proteins, fractalkine and MadCAM (R&D systems, Inc.) were included, both of which are implicated in intestinal pathology. Fractalkine expression is enhanced in inflammatory Crohn’s disease lesions [15] and MadCAM, expressed on the surface of high endothelial venules in the gut, is the ligand for the integrin α4β7 allowing lymphocyte homing to the gut [16]. Recombinant proteins were spotted in triplicate onto two plastic petri dishes at a concentration of 50 μg/ml. Plates were blocked with 3 % BSA, washed and parasite suspension added for 1 h at 37 °C with gentle rotation every 10 min. Unbound parasites were washed off, adhering parasites fixed with 1 % glutaraldehyde followed by staining with 1 % Giemsa in PBS. Images were taken using direct microscopy by *Image*-*Pro Analyser* and adhering parasites counted using *QCapture Pro* (QImaging) software. Results are presented as the mean number of parasites/mm^2^ protein adjusted to 3 % parasitaemia.

For the rosetting assay, 20 μl of parasite suspension in binding medium containing 5 μg/ml acridine orange (Sigma–Aldrich) and 20 % AB serum was rotated for 30 min at room temperature. The rosetting frequency of each isolate was scored by determining the percentage of 200 trophozoite-infected erythrocytes bound to two or more uninfected erythrocytes using a fluorescence microscope (Nikon eclipse 80*i*, Japan) [17].

### Statistical analysis

Continuous variables were compared between groups using the Mann–Whitney U test or Kruskal–Wallis H test. Spearman’s rho correlation coefficient was used to determine a relationship between continuous variables. Statistical analyses were performed using Stata version 12 (StataCorp LP).

## Results

RBCs of 109 children hospitalized with *P. falciparum* malaria were cultured to allow iRBCs to grow to maturity. Of the 109 isolates, iRBCs of 48 (44 %) samples grew to maturity. The high failure rate may be explained by the use of anti-malarials prior to hospital admission, which is commonplace in this region of Uganda. There was no significant difference with respect to haematological and clinical data between the two groups and, therefore, the samples evaluated for adhesion can be considered representative of the overall group of children (Table 1). Of those children whose parasites grew in culture, 79.2 % (n = 38) had severe malaria defined clinically, with almost half (n = 22) suffering from a degree of impaired conscious (prostration or coma), but only 8.3 % (n = 4) had unrousable coma (Blantyre coma score ≤2). In the children with viable parasites, the number of children with endotoxemia (EAA units ≥0.4) was somewhat underrepresented in the group of children with available adhesion data (Chi squared 3.3, p = 0.067).

**Table 1:** Baseline group characteristics

	Children with parasite adhesion data	Children without parasite adhesion data
Number of children	48	61
Median age (months)	25.5 (12–40)	26 (15–44)
Median haemoglobin	6.5 (4.3–9.5)	5.9 (3.8–9.3)
Severe malaria	38 (79.2 %)	49 (80.3 %)
Impaired consciousness	22 (45.8 %)	25 (37.3 %)
Coma	4 (8.3 %)	4 (6.5 %)
Severe anaemia	18 (37.5 %)	27 (44 %)
Respiratory distress	37 (77 %)	43 (70.5 %)
Endotoxemia (EAA ≥ 0.4 U)	16 (33 %)	31 (50.8 %)
I-FABP ≥183 pg/ml	10 (20.8 %)	12 (19.6 %)

**Fig. 1:**
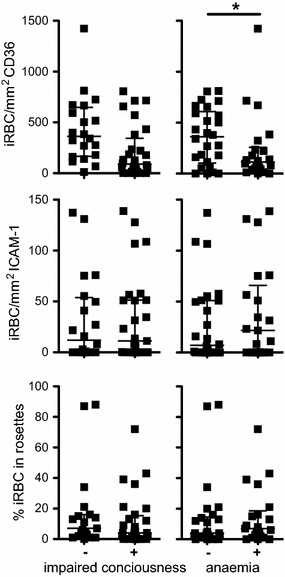
Adhesion of iRBC in children with impaired consciousness or anaemia. Shown are dot plots of the number of iRBCs/mm^2^ binding to CD36, ICAM-1 or the percentage of iRBCs in rosettes with RBCs in children with (*plus*) or without (*minus*) impaired consciousness (prostration or coma) and in children with (*plus*) or without (*minus*) anaemia (Hb ≤ 7). Median and interquartile range are indicated, * denotes p value of <0.05 (Mann–Whitney U test)

As previously described, the majority of parasites demonstrated binding to the endothelial receptors CD36 and to a lesser extent to ICAM-1 [14, 18]. There was no binding observed in any of the samples to fractalkine and MadCAM. There was a significant association between adhesion to CD36 and ICAM-1 (Spearman rho 0.34, p = 0.017) however neither was correlated with rosetting (Spearman rho = 0.09 & 0.07; p = 0.53 and p = 0.64 respectively). Reduced adhesion to CD36 but not adhesion to ICAM-1 or rosetting was associated with malarial anaemia (Spearman rho = 0.41, p = 0.004) (Fig. 1). There was no relationship between binding to ICAM-1 and coma (p = 0.65), however adhesion under static conditions does not have the sensitivity to establish this relationship, which has been demonstrated in vitro under flow [14].

**Fig. 2:**
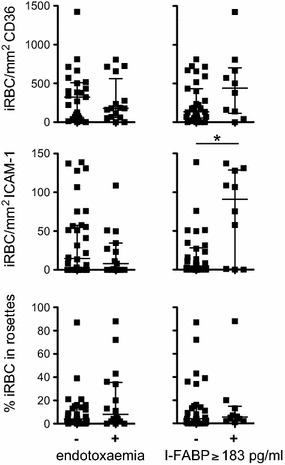
Adhesion of iRBC in children with endotoxemia or increased plasma concentrations of I-FABP. Shown are dot plots of the number of iRBCs/mm^2^ binding to CD36, ICAM-1 or the percentage of iRBCs in rosettes with RBCs in children with (*plus*) or without (*minus*) endotoxemia and in children with (*plus*) or without (*minus*) increased plasma concentrations of I-FABP (I-FABP ≥ 183 ng/ml) indicating acute enterocyte damage. Median ans interquartile range are indicated, * denotes p value of <0.05 (Mann–Whitney U test)

**Fig. 3:**
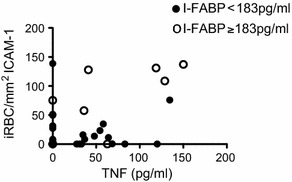
Correlation between iRBC adhesion to ICAM-1 and the plasma concentration of TNF. Shown is a scatter plot of iRBCs/mm^2^ binding to ICAM1 and the plasma concentration of TNF (pg/ml). Values from children with increased plasma concentration of I-FABP (I-FABP ≥ 183 ng/ml) are indicated by an *open circle* and values of children with a plasma concentration below pathological levels (I-FABP < 183 ng/ml) are indicated by *black circles*

Next, an association with adhesion of iRBCs with either marker of intestinal compromise was investigated: endotoxemia (EAA ≥0.4) and a marker of acute enterocyte damage (I-FABP ≥183 pg/ml plasma). Adhesion of iRBCs to CD36 and ICAM-1 as well as rosetting was similar in children with or without endotoxemia (Fig. 2). By contrast, adhesion of iRBCs to ICAM-1 but not CD36 or rosetting was significantly increased in children who had elevated plasma concentrations of I-FABP (Spearman rho = 0.36, p = 0.022) (Fig. 2). Samples with increased binding to ICAM-1 were also associated with elevated plasma concentrations of TNF (Spearman rho = 0.30, p = 0.038) particularly in children who also had raised levels of I-FABP (Spearman rho = 0.62, p = 0.055) although here the difference did not quite reach significance (Fig. 3).

## Discussion

In children with malaria and acute gut injury, adhesion of mature iRBCs to ICAM-1 under static conditions was significantly increased. These data suggest that acute enterocyte damage is associated with the binding of sequestered iRBCs to ICAM-1 expressed on the surface of gut endothelial cells. Without a healthy enterocyte barrier, it follows that intestinal pathogens or their products such as endotoxin may pass unimpeded into the systemic circulation further complicating malaria infection.

There is a well-established link between adhesion of iRBCs to ICAM-1 and cerebral malaria. Histological examination of brains of patients who died of cerebral malaria have shown that iRBCs co-localized with ICAM-1 [19] and binding to ICAM-1 was higher in children with cerebral malaria [14, 18]. Interestingly, in children with cerebral malaria, sequestration of iRBCs is high in the gut as well as the brain compared to children who died with parasitaemia and other infections [20]. Sequestration in both brain and gut tissue may, therefore, be driven by expression of ICAM-1. ICAM-1 is constitutively expressed on endothelial cells in the gut microvasculature and neutrophils from blood of malaria patients cause endothelial damage in vitro by binding to ICAM-1 [21]. However, it remains difficult to determine whether ICAM-1 expression is itself causative or merely a by-product of local inflammation and an altered gastrointestinal barrier. Whilst ICAM-1 expression increases pro-inflammatory cytokines via adhesion of iRBCs [22], pro-inflammatory cytokines can also up regulate ICAM-1 expression in the absence of parasite adhesion [23].

Overall, the data presented here correspond with other published studies on the binding of parasitized RBCs under static conditions [18]; the majority of iRBC isolates bound to CD36 and a lesser number to ICAM-1. Moreover the finding of reduced binding to CD36 in malarial anaemia was reproduced as described previously [18, 24].

This study is the first to examine in vitro the role of parasite adhesion in malaria subjects with evidence of intestinal compromise. I-FABP, a small water-soluble protein present in mature enterocytes, is abundantly expressed on villous tips in the small intestine and is therefore a useful marker of acute enterocyte damage. It has been used to investigate intestinal damage in other cohorts including children undergoing surgery for congenital heart disease and neonates with suspected necrotising enterocolitis and meningococcal septicaemia where it can delineate between early sepsis and specific gut damage [25–27]. In this cohort, all infants were unwell enough to warrant admission to hospital with malaria. Almost 80 % had severe malaria and signs of at least one life threatening clinical syndrome attesting to the severity of disease. It is possible that the extent of intestinal damage in this cohort was underestimated due to the short half-life of I-FABP. This may explain the lack of correlation between endotoxemia and elevated I-FABP. In addition, although I-FABP does not provide a measure of microbial translocation, it may be a more specific marker of gut injury than endotoxin. Its relative abundance on villous tips is the same site where parasite sequestration has been observed to be most intense [11]. Furthermore, endotoxin levels can be difficult to accurately measure and can be elevated for a number of other reasons [28].

There were several limitations to this study. First, the high fall out rate when thawing and culturing iRBCs, comparable with other adhesion studies [14], halved the sample size. An important factor may have been the use of artemisinin-based combination anti-malarial therapies prior to hospital admission, indicating successful role out of the recent changes in first line management in Uganda, since their introduction in 2009—which will have implications for studies of fresh parasite isolates in future. Second, the case report form used in the FEAST trial did not document in detail clinical information regarding gastrointestinal symptoms and blood cultures were not routinely taken, which would have enhanced our definition and description of gastrointestinal compromise. Thirdly, the in vitro experiment lacked specificity to the gut. The attempt to introduce other gut specific recombinant proteins such as MadCAM was unsuccessful and, therefore, the study is based on the assumption that adhesion in the gut is similar to elsewhere in the body. Without a gut-specific model to assess adhesion in vitro to gut endothelium, it will be difficult to tease apart the precise contribution of iRBC-ICAM1 binding to intestinal compromise in patients with malaria. This warrants further exploration.

These findings lend further evidence to a link between cytoadhesion and intestinal damage mediated by the sequestration of iRBCs in gut microvasculature. However there are other pathways that may also contribute to gastrointestinal damage in the context of malaria. Systemic inflammation, which is associated with severe disease, but also a hallmark of the sepsis syndrome can lead to gut epithelial cell damage [29]. The correlation between the pro-inflammatory cytokine TNF and elevated I-FABP levels which corroborates this [5]. In addition, malarial anaemia drives systemic inflammation but can also precipitate end organ damage via splanchnic hypoperfusion especially in combination with shock. However, an association between either anaemia or signs of hypoperfusion and markers of intestinal damage was not observed.

## Conclusions

In conclusion, the increased parasite adhesion to ICAM-1 in children with evidence of intestinal damage is an important finding, which highlights the complex aetiology behind increased susceptibility to gram-negative bacteraemia in children with malaria. Further research is required to unpick the relative contribution of iRBC sequestration and ICAM-1 binding towards gut barrier compromise. Only then the precise sequence of events in the pathogenesis of malaria-IBI co-infection and progress towards adjunctive therapies including antibiotics can be understood.

## Abbreviations

iRBCs: infected red blood cells
I-FABP: intestinal fatty acid binding protein
EGNO: enteric gram-negative organisms
IBI: invasive bacterial infection

## References

[1] Church J, Maitland K (2014). Invasive bacterial co-infection in African children with *Plasmodium falciparum* malaria: a systematic review. BMC Med.

[2] Biggs HM, Lester R, Nadjm B, Mtove G, Todd JE, Kinabo GD (2014). Invasive Salmonella infections in areas of high and low malaria transmission intensity in Tanzania. Clin Infect Dis.

[3] Wilairatana P, Meddings JB, Ho M, Vannaphan S, Looareesuwan S (1997). Increased gastrointestinal permeability in patients with *Plasmodium falciparum* malaria. Clin Infect Dis.

[4] Sowunmi A, Ogundahunsi OA, Falade CO, Gbotosho GO, Oduola AM (2000). Gastrointestinal manifestations of acute falciparum malaria in children. Acta Trop.

[5] Olupot-Olupot P, Urban BC, Jemutai J, Nteziyaremye J, Fanjo HM, Karanja H (2013). Endotoxemia is common in children with *Plasmodium falciparum* malaria. BMC Infect Dis.

[6] Marshall JC, Foster D, Vincent JL, Cook DJ, Cohen J, Dellinger RP (2004). Diagnostic and prognostic implications of endotoxemia in critical illness: results of the MEDIC study. J Infect Dis.

[7] Ikeda T, Ikeda K, Suda S, Ueno T (2014). Usefulness of the endotoxin activity assay as a biomarker to assess the severity of endotoxemia in critically ill patients. Innate Immun.

[8] Holland J, Carey M, Hughes N, Sweeney K, Byrne PJ, Healy M (2005). Intraoperative splanchnic hypoperfusion, increased intestinal permeability, down-regulation of monocyte class II major histocompatibility complex expression, exaggerated acute phase response, and sepsis. Am J Surg.

[9] Hietbrink F, Besselink MG, Renooij W, de Smet MB, Draisma A, van der Hoeven H (2009). Systemic inflammation increases intestinal permeability during experimental human endotoxemia. Shock.

[10] Chau JY, Tiffany CM, Nimishakavi S, Lawrence JA, Pakpour N, Mooney JP (2013). Malaria-associated l-arginine deficiency induces mast cell-associated disruption to intestinal barrier defenses against nontyphoidal *Salmonella* bacteremia. Infect Immun.

[11] Pongponratn E, Riganti M, Punpoowong B, Aikawa M (1991). Microvascular sequestration of parasitized erythrocytes in human falciparum malaria: a pathological study. Am J Trop Med Hyg.

[12] Seydel KB, Milner DA, Kamiza SB, Molyneux ME, Taylor TE (2006). The distribution and intensity of parasite sequestration in comatose Malawian children. J Infect Dis.

[13] Maitland K, Kiguli S, Opoka RO, Engoru C, Olupot-Olupot P, Akech SO (2011). Mortality after fluid bolus in African children with severe infection. N Engl J Med.

[14] Ochola LB, Siddondo BR, Ocholla H, Nkya S, Kimani EN, Williams TN (2011). Specific receptor usage in *Plasmodium falciparum* cytoadherence is associated with disease outcome. PLoS One.

[15] Brand S, Hofbauer K, Dambacher J, Schnitzler F, Staudinger T, Pfennig S (2006). Increased expression of the chemokine fractalkine in Crohn’s disease and association of the fractalkine receptor T280M polymorphism with a fibrostenosing disease phenotype. Am J Gastroenterol.

[16] Shaw SK, Brenner MB (1995). The beta 7 integrins in mucosal homing and retention. Semin Immunol.

[17] Warimwe GM, Fegan G, Musyoki JN, Newton CR, Opiyo M, Githinji G (2012). Prognostic indicators of life-threatening malaria are associated with distinct parasite variant antigen profiles. Sci Transl Med.

[18] Newbold C, Warn P, Black G, Berendt A, Craig A, Snow B (1997). Receptor-specific adhesion and clinical disease in *Plasmodium falciparum*. Am J Trop Med Hyg.

[19] Turner GD, Morrison H, Jones M, Davis TM, Looareesuwan S, Buley ID (1994). An immunohistochemical study of the pathology of fatal malaria. Evidence for widespread endothelial activation and a potential role for intercellular adhesion molecule-1 in cerebral sequestration. Am J Pathol.

[20] Montgomery J, Milner DA, Tse MT, Njobvu A, Kayira K, Dzamalala CP (2006). Genetic analysis of circulating and sequestered populations of *Plasmodium falciparum* in fatal pediatric malaria. J Infect Dis.

[21] Hemmer CJ, Vogt A, Unverricht M, Krause R, Lademann M, Reisinger EC (2008). Malaria and bacterial sepsis: similar mechanisms of endothelial apoptosis and its prevention in vitro. Crit Care Med.

[22] Wu Y, Szestak T, Stins M, Craig AG (2011). Amplification of *P*. *falciparum* Cytoadherence through induction of a pro-adhesive state in host endothelium. PLoS One.

[23] Goebel S, Huang M, Davis WC, Jennings M, Siahaan TJ, Alexander JS (2006). VEGF-A stimulation of leukocyte adhesion to colonic microvascular endothelium: implications for inflammatory bowel disease. Am J Physiol Gastrointest Liver Physiol.

[24] Rogerson SJ, Tembenu R, Dobano C, Plitt S, Taylor TE, Molyneux ME (1999). Cytoadherence characteristics of *Plasmodium falciparum*-infected erythrocytes from Malawian children with severe and uncomplicated malaria. Am J Trop Med Hyg.

[25] Schurink M, Scholten IG, Kooi EM, Hulzebos CV, Kox RG, Groen H (2014). Intestinal fatty acid-binding protein in neonates with imminent necrotizing enterocolitis. Neonatology.

[26] Pathan N, Burmester M, Adamovic T, Berk M, Ng KW, Betts H (2011). Intestinal injury and endotoxemia in children undergoing surgery for congenital heart disease. Am J Respir Crit Care Med.

[27] Derikx JP, Bijker EM, Vos GD, van Bijnen AA, Heineman E, Buurman WA (2010). Gut mucosal cell damage in meningococcal sepsis in children: relation with clinical outcome. Crit Care Med.

[28] Erridge C, Attina T, Spickett CM, Webb DJ (2007). A high-fat meal induces low-grade endotoxemia: evidence of a novel mechanism of postprandial inflammation. Am J Clin Nutr.

[29] Cavaillon JM, Adib-Conquy M (2006). Bench-to-bedside review: endotoxin tolerance as a model of leukocyte reprogramming in sepsis. Crit Care.

